# Evaluation of the Anticancer and Biological Activities of Istaroxime via Ex Vivo Analyses, Molecular Docking and Conceptual Density Functional Theory Computations

**DOI:** 10.3390/molecules28227458

**Published:** 2023-11-07

**Authors:** Ege Gok, Naz Unal, Burcin Gungor, Gulderen Karakus, Savas Kaya, Pakize Canturk, Konstantin P. Katin

**Affiliations:** 1 Department of Pharmaceutical Biotechnology, Faculty of Pharmacy, Sivas Cumhuriyet University, 58140 Sivas, Turkey; ecz.egegok@gmail.com; 2 Department of Biochemistry, Faculty of Pharmacy, Yeditepe University, 34755 Istanbul, Turkey; nazunal2012@gmail.com (N.U.); burcin.gungor@yeditepe.edu.tr (B.G.); 3 Department of Pharmaceutical Basic Sciences, Faculty of Pharmacy, Sivas Cumhuriyet University, 58140 Sivas, Turkey; gulderen@cumhuriyet.edu.tr; 4 Department of Chemistry, Faculty of Science, Sivas Cumhuriyet University, 58140 Sivas, Turkey; 5 Nanoengineering in Electronics, Spintronics and Photonics Institute, National Research Nuclear University MEPhI, 115409 Moscow, Russia; kpkatin@yandex.ru

**Keywords:** Istaroxime, topoisomerase enzymes, anticancer drugs, Density Functional Theory

## Abstract

Cancer is a disease that occurs as a result of abnormal or uncontrolled growth of cells due to DNA damage, among many other causes. Certain cancer treatments aim to increase the excess of DNA breaks to such an extent that they cannot escape from the general mechanism of cell checkpoints, leading to the apoptosis of mutant cells. In this study, one of the Sarco-endoplasmic reticulum Ca^2+^ATPase (SERCA2a) inhibitors, Istaroxime, was investigated. There has been very limited number of articles so far reporting Istaroxime’s anticancer activity; thus, we aimed to evaluate the anticancer effects of Istaroxime by cell proliferation assay and revealed the cytotoxic activity of the compound. We further determined the interaction of Istaroxime with topoisomerase enzymes through enzyme activity tests and detailed molecular modeling analysis. Istaroxime exhibited an antiproliferative effect on A549, MCF7, and PC3 cell lines and inhibited Topoisomerase I, suggesting that Istaroxime can act as a Topoisomerase I inhibitor under in vitro conditions. Molecular docking analysis supported the experimental observations. A chemical reactivity analysis of the Istaroxime molecule was made in the light of Density Functional Theory computations. For this aim, important chemical reactivity descriptors such as hardness, electronegativity, and electrophilicity were computed and discussed as detailed.

## 1. Introduction

The ability to estimate and understand interactions between proteins and small molecules holds supreme importance in the realm of biology and drug development. This predictive process empowers scientists to delve deep into the intricacies of biological processes, offering a profound comprehension that serves as the bedrock for advancing pharmaceutical research and innovation. By accurately foretelling these interactions, researchers gain access to a treasure trove of insights within the labyrinthine world of biology. They can discern the nuanced relationships between proteins and small molecules, shedding light on the molecular ballet that orchestrates various cellular activities. Moreover, this foresight allows scientists to identify potential targets for therapeutic interventions, opening doors to the development of novel drugs and treatments. The significance of this predictive ability reverberates throughout the scientific community. It serves as the linchpin in unraveling the multifaceted tapestry of cellular mechanisms. Whether it be elucidating the intricacies of cellular signaling pathways, deciphering the fine-tuned mechanisms of protein regulation, or unraveling the convoluted pathways underlying various diseases, predictive modeling of protein-small molecule interactions plays a pivotal role in advancing our understanding of these complex biological processes. In essence, it acts as a guiding light, illuminating the way forward in the quest for new therapies and breakthroughs in the field of medicine [1,2]. Furthermore, precision in predicting interactions between proteins and small molecules constitutes a fundamental requirement in the realm of drug development. This precision equips researchers with the tools they need to pinpoint potential drug targets and enhance the formulation of novel medications. The grasp of these interactions empowers scientists to exert control over specific biological pathways, paving the way for the creation of highly tailored drugs that can precisely fine-tune protein activities to achieve therapeutic goals.

Many living things on earth, even almost all living things, have a magic wand known as topoisomerase enzymes that preserve their vitality by cutting their DNA molecules into pieces and then reassembling them. Considering that DNA molecules are stored inside tiny cells, coiled and entangled over histone proteins, thanks to this magic wand, the DNA molecule can be cut in the vital intracellular events and processes in a controlled manner, and the essential processes can be completed without damaging the DNA [3,4,5]. The ability of the DNA molecule to be replicated and/or transcribed in a practical and intact state, and even the formation of a cellular response to many different anticancer drugs, is achieved by the coordinated activity of many different topoisomerase enzymes by decreasing or increasing the expression of these enzymes. As it is known that topoisomerase enzymes have been targeted by anticancer drugs for a long time, it is also known that most of the molecules that inhibit these enzymes are both effectively lethal and toxic in cancer cells and negatively affect the quality of life of patients. Despite this toxicity, inhibition of the activities of these enzymes remains in medical use and remains popular in the development of anticancer compounds [6]. Less toxic, more stable topoisomerase enzyme inhibitor derivatives are also under investigation. In fact, many of these derivatives have led to successful results in cancer types that have been difficult to treat until recently. Because cancerous cells and dividing cells rapidly replicate DNA molecules, mechanically inhibiting the hard-working nature of topoisomerase enzymes makes it a very logical approach to cancer treatment. 

Clinical Topoisomerase I inhibitors derived from Camptothecin, such as Irinotecan and Topotecan, appear to be highly effective in treating many types of cancer, including colon, ovarian, pancreatic, and small-cell lung cancers [7,8,9]. The selective use of DNA-damaging molecules is a very common choice for rapidly proliferating cancer cells. Thus, damage to DNA can cause the death of cancer cells by blocking replication through many intracellular pathways [10,11]. DNA topoisomerase inhibitors stand out in the field of anticancer drug development within the scope of this approach. With the inhibitory activity of these molecules, the cell’s chance to relieve stress provided by topoisomerases is prevented, and DNA-drug-enzyme complexes are formed in the cell, which will split the DNA strands into lethal fragments [12,13]. While Topoisomerase I enzymes create single-strand breaks in DNA, Topoisomerase II enzymes create double-strand breaks in DNA, thus reducing the torsional stress on DNA and then allowing the DNA strands to relax from their formerly tense state [9,14]. The breaks in these DNA strands are mostly reversible, with topoisomerase enzymes rejoining the strands in a controlled manner [3,11,14].

Sercoplasmic endoplasmic Ca^2+^ATPase (SERCA) shows promise as a potential anti-cancer target due to its important role in cellular Ca^2+^ regulation, cell proliferation, and apoptosis. As one of the most potent Ca^2+^ATPase inhibitors, Istaroxime (IST) has yet to be elucidated in cancer treatment. IST appears to be effective in cancer treatments, and more specifically, it has been reported to be effective against prostate cancer [15], whereas demonstration of its potential anticancer activities has been limited to a few articles in cancer research. Considering the cardiological side effects of anti-cancer agents, it is thought that the chemical Istaroxime can be used in combinational treatment approaches [16], and moreover, Istaroxime is still a new by molecule proven to treat heart diseases safely and is gaining interest in a constant manner [17]. 

Istaroxime (IST) is a compound under investigation for use in acute heart failure, derived from androstenedione [18]. There have been reports indicating that IST inhibits sodium/potassium (Na-K) ATPase and stimulates the calcium ATPase isoform 2a (SERCA2a) in patients with acute heart failure. IST was shown to be safer than currently available treatments for acute heart failure (AHF), and yet the safety and efficacy of IST are still under evaluation for clinical trials for treatments for AHF [19,20]. The compound is considered a good option for treating patients with high-risk conditions, as it allows for faster stabilization and earlier administration of other life-saving treatments [21]. In a phase 2a study, IST was reported to improve heart failure-related blood pressure and echocardiographic measurements in patients with AHF-related precardiogenic shock [22]. 

Given the anti-neoplastic potential of Na^+^/K^+^ ATPase inhibitors, the anti-neoplastic activity of IST in different cancer cell lines and in vivo tumor growth reduction in prostate cancer xenograft models are of interest. This raises the question of whether this molecule has an anticancer effect through different pathways or even in different types of cancer. IST has been shown to effectively reduce survival at low molecular doses in lung, melanoma, ovarian, renal, CNS, breast, pancreas, colon, and prostate cancer cell lines [15]. Moreover, according to the results of the same study, PC3 and DU145 prostate cancer cells were the most sensitive ones to IST treatment, and the compound suppressed c-Myc oncoprotein expression, induced apoptosis and caspase-3 activation in prostate cancer cells, and inhibited cell migration in DU145 cells [15,18,23]. In our study, we wanted to further evaluate the anticancer effects of IST and examined its cytotoxic and anticancer properties. We analyzed the IC50 values of the IST molecule in lung, prostate, and breast cancer cell lines by cell proliferation assay. We then examined the interactions of the IST molecule with topoisomerase enzymes at the molecular level and sophistically evaluated in silico analyzes of compound-enzyme interactions.

### Details of the Conceptual Density Functional Theory Based Computations

Conceptual Density Functional Theory (CDFT) found important applications in the studies, including the local and global reactivity analysis of molecular systems, in the chemical reactivity-based branch of Density Functional Theory introduced by Kohn. Here, the chemical reactivity of the IST molecule is analyzed with the help of quantum chemical parameters and the electronic structural rules of CDFT. In the CDFT, well-known quantum chemical descriptors like chemical potential (*µ*), electronegativity (*χ*), hardness (*η*), and softness (*σ*) are presented through the following mathematical relations [24,25].

$$\mu =-\chi ={\left[\frac{\partial E}{\partial N}\right]}_{\nu (r)}\;\eta ={\left[\frac{\partial^{2}E}{\partial N^{2}}\right]}_{\nu (r)}\;\sigma =1/\eta$$

It can be seen from these relations that chemical potential and chemical hardness are defined as first and second derivatives with respect to the total number of electrons (*N*) of the total electronic energy (*E*) at a constant external potential. 

The first electrophilicity index (*ω*_1_) proposed by Parr, Szentpaly, and Liu [26] is based on the electronegativity and chemical hardness of atomic and molecular systems and is given as:

$$\omega_{1}=\chi^{2}/2\eta =\mu^{2}/2\eta$$

By applying the finite difference approach to the forgiven mathematical relations, the equations based on ground state ionization energy (*I*) and electron affinity (*A*) of chemical potential, electronegativity, hardness, and softness are obtained [27].

$$\mu =-\chi =-\left(\frac{I+A}{2}\right)\;\eta =I-A\;\sigma =1/(I-A)$$

As a result of the theoretical derivation process performed by Gazquez and coworkers [28], for the prediction of the electrodonating power (*ω*^−^) and electroaccepting power (*ω*^+^) of the molecules, the following formulae were obtained:

$$\omega^{-}={\left(3I+A\right)}^{2}/(16(I-A))\;\omega^{+}={\left(I+3A\right)}^{2}/(16(I-A))$$

The vertical ground state ionization (*I*) energy and electron affinity (*A*) of a molecule were calculated from the single point energies of anionic and cationic forms [29]: 

*I* = *E*(cationic form) − *E*(neutral)

*A* = *E*(neutral) − *E*(anionic form)

## 2. Results

### 2.1. The Cell Proliferation Assay

**Figure 1:**
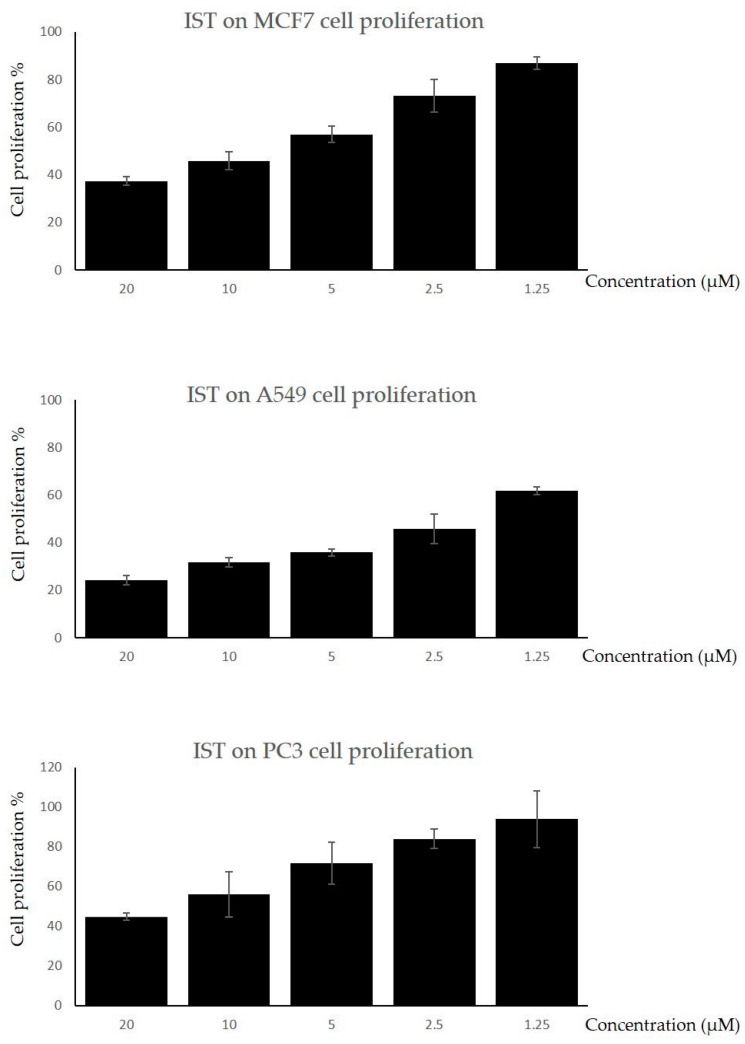
The effect of IST on cancer cell proliferation. MCF7, A549, and PC3 cell lines were treated with IST at the indicated concentrations for 72 h. A MTT test was performed to determine the cytotoxic effect of IST. For each panel, the group of cells that were not administered to IST was considered to exhibit 100% proliferation. Each condition was studied in 5 replicates. Mean ± std.

The anti-proliferative effect of IST was studied on MCF7, A549, and PC3 cell lines. Each cell line grown in a 96-well plate was exposed to IST’s five different concentrations (1.25–20 µM) for 72 h. MTT assay results demonstrated a concentration-dependent inhibitory effect of IST (Figure 1). The cytotoxic effect of IST was calculated by determining the inhibitory concentrations (IC50) of the compound for each cell line exposed. The IC50 values of IST for MCF7, A549, and PC3 were obtained as 12 µM (R^2^: 0.86), 2 µM (R^2^: 0.88), and 16 µM (R^2^: 0.88), respectively. Accordingly, IST exhibited an anti-proliferative effect on all cell types studied. Of those, the A549 cell line was the most sensitive cell type to IST exposure, representing a very low value of IC50 concentration (Figure 1).

### 2.2. Docking Analyses and CDFT Computations 

**Figure 2:**
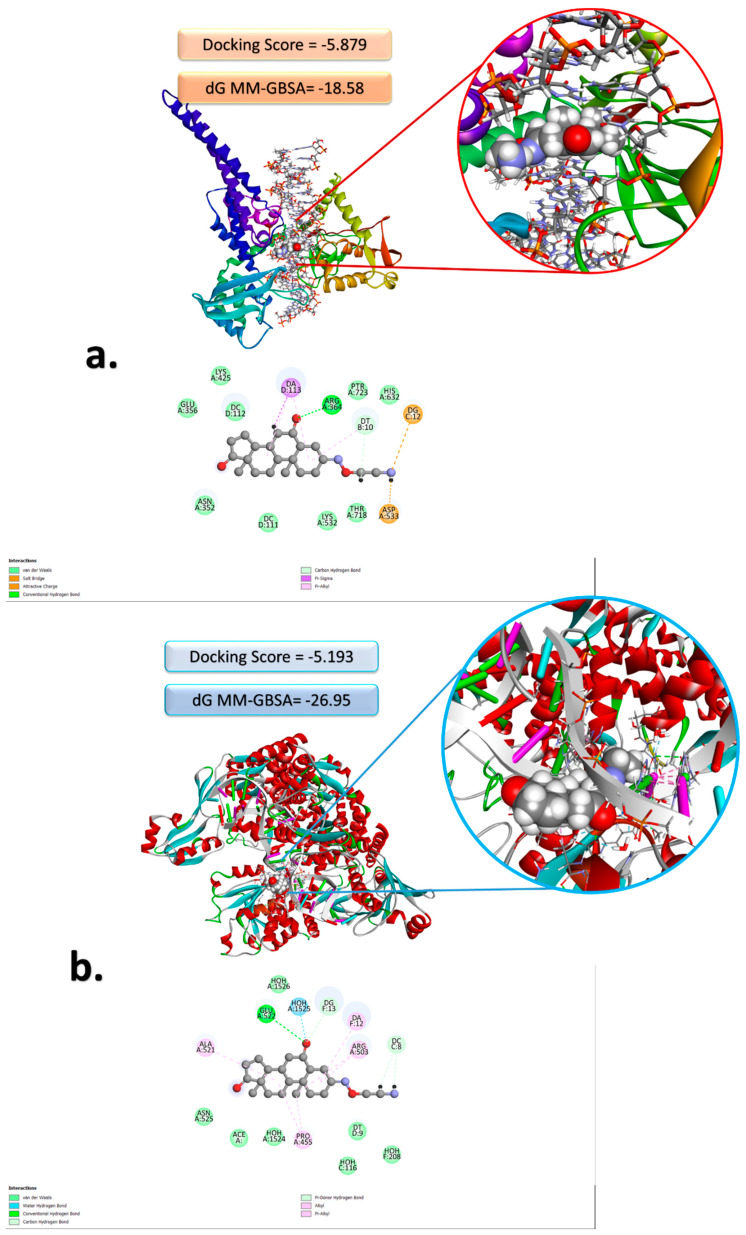
Corresponding 3D and 2D docking poses have the best docking scores for the interaction of the Istaroxime drug with (**a**) Topoisomerase I and (**b**) Topoisomerase II proteins.

**Figure 3:**
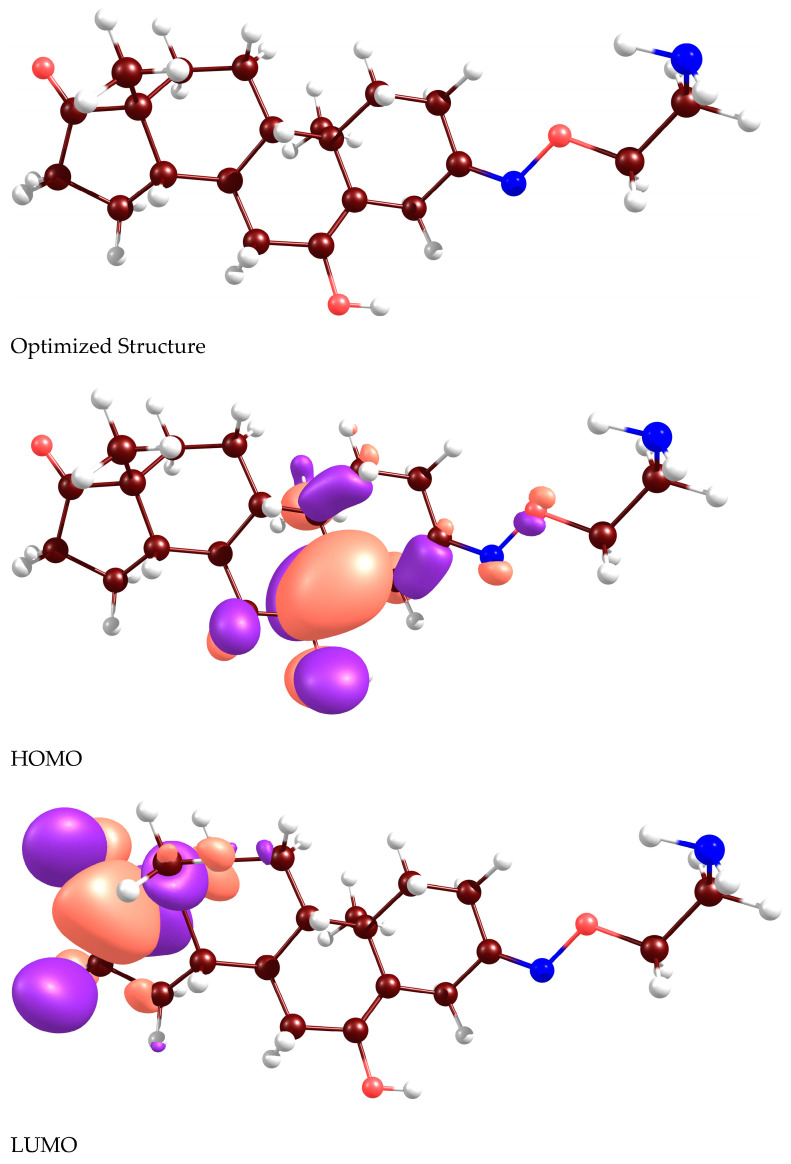
Optimized structure, HOMO, and LUMO images of Istaroxime molecules obtained at the B3LYP/6-311G** level of theory. White, brown, blue, and red balls represent hydrogen, carbon, nitrogen, and oxygen atoms, respectively.

The compelling nature of these findings is further fortified by the drug’s ability to form hydrogen bonds with amino acid side chains during the docking process, a feature that aligns seamlessly with the exceptionally favorable docking score values observed [30,31]. Figure 2 graphically illustrates the drug’s strong binding affinity to the protein pocket, underscoring a noteworthy docking score of approximately −5 kcal/mol for both protein structures. Additionally, it showcases remarkably high values of dG, measuring −26.95 for Topoisomerase II and −18.25 kcal/mol for Topoisomerase I. This remarkable interaction between the molecule and the target proteins hinges on its intrinsic capacity to establish hydrogen bonds, a phenomenon facilitated by the electron pair located at the oxygen atoms and the amino group of the drug. Furthermore, the molecule actively participates in π-alkyl interactions by virtue of its aromatic rings. In essence, these results not only highlight Istaroxime’s potential as a potent drug candidate but also shed light on the molecular intricacies underpinning its strong binding to specific protein targets, ultimately paving the way for further exploration and development in the field of pharmaceutical research. In Figure 2, 2D and 3D structures obtained via docking analysis regarding the interaction with topoisomerase I and topoisomerase II of the Istaroxime molecule are presented. Docking scores determined about the interaction with topoisomerase I and topoisomerase II of the studied molecular system are −5.879 and −5.193, respectively. 

In Figure 3, optimized structure, HOMO and LUMO images obtained for the Istaroxime molecule are given. Density Functional Theory computations regarding the mentioned molecular system were done with the B3LYP/6-311G** computational level and using GAMESS-US software (version R1, released 1 May 2013) [32]. In the calculations made, HOMO and LUMO orbital energies were predicted as −5.78 eV and −0.70 eV, respectively. Dipole moment value of the molecule is 0.41 Debye.

Ionization energy and electron affinity of the Istaroxime molecule are 5.75 eV and 0.56 eV, respectively. Note that both values are very close to –HOMO and –LUMO, respectively, in accordance with the Koopmans theorem. One of the most popular parameters of chemical reactivity analysis studies is chemical hardness [33,34,35]. According to the Maximum Hardness Principle [36,37], “It seems to be a rule of nature that molecules arrange themselves so as to be as hard as possible.” As it can be understood from this explanation, hardness is a good indicator of chemical stability. The chemical hardness value of Istaroxime is 5.19 eV. This value supports the idea that Istaroxime is a molecule with high stability. Electronegativity [38] reflects the electron withdrawal power of the chemical species. The electronegativity value of the studied molecule was calculated as: 3.155 eV.

The Minimum Electrophilicity Principle [39] states that electrophilicity is minimized in stable states. Calculated electrophilicity index, electrodonating power (*ω*^−^), and electroaccepting power (*ω*^+^) of the Istaroxime molecule are 0.96, 3.81, and 0.66 eV, respectively. These values characterize quantitatively the ability of the molecule to lose or gain electrons and define its reactivity. 

### 2.3. Enyzme Activity Assays

#### 2.3.1. Supercoiled DNA Relaxation Analyses

**Figure 4:**
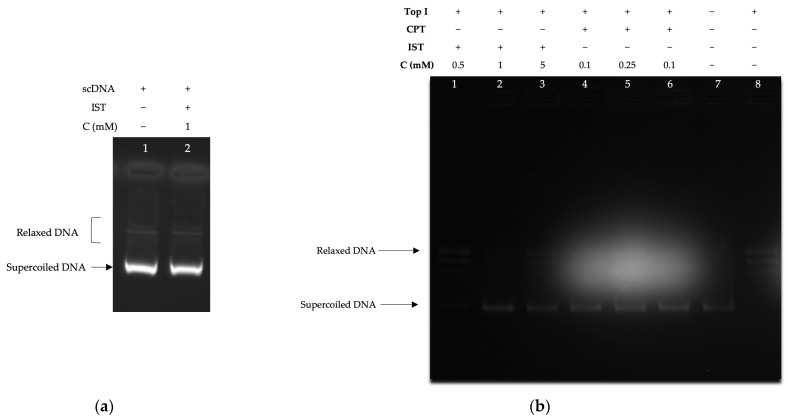
The inhibition profile of IST on topoisomerase I. (**a**) DNA intercalation test on IST. Lanes: all in the presence of 0.5 µg supercoiled DNA, 1; 0.5 µg supercoiled DNA, 2; IST (1 mM). (**b**) IST inhibits the topoisomerase I enzyme. All in the presence of 0.5 μg pBR322 supercoiled DNA, lane 1; 0.5 mM IST in the presence of 1 unit of topoisomerase I, 2; 1 mM IST in the presence of 1 unit of topoisomerase I, 3; 5 mM IST in the presence of 1 unit of topoisomerase I, 4; 0.1 mM CPT in the presence of 1 unit of topoisomerase I, line 5; 0.25 mM CPT in the presence of 1 unit of topoisomerase I, 6; 0.1 mM CPT in the presence of 1 unit of topoisomerase I, 7; 0.5 μg pBR322 supercoiled DNA, 8; 1 unit of topoisomerase I and 0.5 μg pBR322 supercoiled DNA.

To test whether IST has intercalative effects on supercoiled DNA, it was applied to DNA substrates without enzymes in the environment within the conditions of the supercoil relaxation tests [40,41]. According to the results obtained, the compound did not show an intercalative effect (Figure 4a). The effect of IST on topoisomerase I is studied in three different concentrations (0.5 mM, 1 mM, and 5 mM). Results demonstrated that IST inhibited the supercoil relaxation activity of the topoisomerase I enzyme in a concentration-dependent manner. Complete inhibition was observed in the presence of 1 mM IST (Figure 4b). To compare the effect of IST in assays, we administered CPT, a well-known topoisomerase I inhibitor. Results showed that the similar inhibitory fashion of CPT inhibited topoisomerase I at a low concentration (0.1 mM), as expected. 

#### 2.3.2. Decatenation Analyses

**Figure 5:**
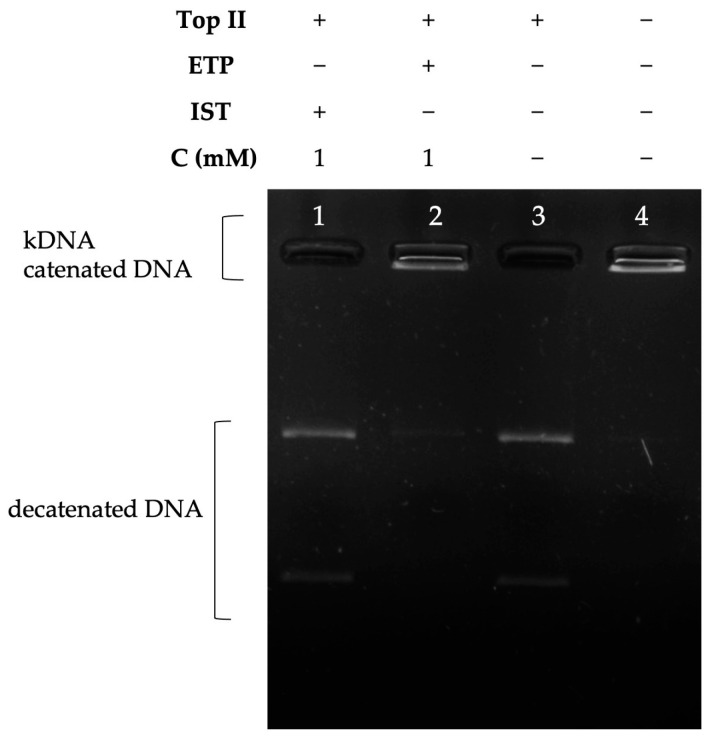
The inhibition profile of IST on topoisomerase II. Lane 1: kDNA in the presence of topoisomerase II and IST at 1 mM concentration; 2: kDNA in the presence of topoisomerase II and ETP at 1 mM concentration; 3: kDNA in the presence of topoisomerase II; and 4: kDNA in the absence of topoisomerase II.

The decatenation assay is a prominent method for selecting drugs capable of inhibiting topoisomerase II in that the DNA substrates used are double-stranded DNA in an interlocked state, thus an assay with a scope reminiscent of human topoisomerase II enzyme activity. Structural differences between decatened products and catenated DNA, which are formed due to enzyme inhibition, help to identify topoisomerase II inhibitors [41]. In our study, we analyzed the inhibition effect of the Istaroxime compound on the topoisomerase II enzyme. According to our results, IST did not act as a topoisomerase II enzyme inhibitor at a concentration of 1 mM (Figure 5), while Etoposide, a well-known topoisomerase II inhibitor, inhibited topoisomerase II at a concentration of 1 mM (Figure 5).

## 3. Discussion

The gene transfer and information exchange of living organisms are carried out by DNA replication with the help of topoisomerase enzymes; thus, DNA topoisomerase enzymes are of great importance for the survival of living organisms [42]. As long as our DNA molecules remain as intact as possible throughout life, we can remain quite healthy with the flawless functioning of various metabolic events in our body. On the other hand, in diseases such as cancer, DNA molecules multiply rapidly, tend to lengthen their telomeres, and eventually terminate the life they have been holding together. For this reason, topoisomerases have become the target of many cancer drugs [43,44]. Reasons of targeting topoisomerase I enzyme, are remarkable findings like rapidly dividing cancer cells with higher topoisomerase I levels being sensitive to topoisomerase I inhibitors, and regarding the significance of the cellular activities of topoisomerase enzymes in cells, one can assume that many apoptotic pathways can be activated, whether the inhibition of topoisomerase enzymes is a priority of an anticancer drug [44,45,46].

The FDA-approved CPT derivatives topotecan and irinotecan have demonstrated the potential for use of topoisomerase I inhibitors and have led to the importance of targeting topoisomerase I in the treatment of cancers that rely on the function of topoisomerase I for survival, such as highly malignant tumor cells [44,45,46,47]. 

Many traditional chemotherapeutics and some new anti-cancer signaling inhibitors carry the risk of heart dysfunction and many important cardiovascular side effects, such as heart failure and arterial hypertension, due to their mechanism of action. Some of these side effects cause irreversible and progressive cardiovascular disease, and it is difficult to strike a balance between the need for cancer treatment and the risk assessment from cancer drug-related cardiovascular side effects to prevent long-term damage [16]. In this study, one of the sarco-endoplasmic reticulum Ca^2+^ATPase (SERCA2a) inhibitors, IST, was investigated. The compound caught our attention by having antitumor activity in in vitro and in vivo models [15]. In addressing this compound, there are notable factors like having a 2a isomerism as well as being a Sercoplasmic endoplasmic reticulum calcium ATPase inhibitor. Sercoplasmic endoplasmic Ca^2+^ATPase (SERCA) shows promise as a potential anti-cancer target and further elucidating the importance of SERCA, there is growing interest in the role of SERCA in diseases such as cancer due to its important role in maintaining intracellular Ca^2+^ levels within acceptable limits to prevent Ca^2+^-mediated autophagy and apoptosis [48,49,50]. As a result of the fact that some cancer cells appear to be more prone to Ca^2+^-mediated apoptosis, an approach to the development of thapsigargin-based anticancer drugs has been followed. A thapsigargin prodrug containing a peptide that targets prostate-specific proteases is currently being evaluated as a potential cancer chemotherapeutic agent for the treatment of prostate cancer [49,51]. Although the Ca^2+^ ATPase (SERCA) inhibitory activity of Istaroxime has been sufficiently proven, there are only a handful of studies in the literature proving its anticancer activities. IST was reported as a potential anticancer agent in 2016 and since then, its anticancer activities did not come to an attention. IST was reported to be a potential anticancer agent in 2016 [15], and since then, IST was more of interest for cardiac research [52]. We argue that the fact that the phase studies on the use of IST in heart diseases continue with a priority is the most common reason for not investigating the anticancer activities of IST with a sufficient number of publications, and we think that the lack of publications on this subject is supported by our study. 

In our study, we measured the IC 50 values of IST on three different cancer cell line. Ex vivo enzyme activities were tested on topoisomerase I and topoisomerase II, and as a promising fact, IST selectively showed an inhibition effect on topoisomerase I. Directed by the disadvantages of the high cardiological toxic effects of anti-cancer drugs used today, researchers are prompted to search for more effective and less toxic drugs with fewer side effects. Since more effective molecules have not yet taken their place, drugs that inhibit topoisomerases are frequently used in cancer treatment because of their proven anti-cancer activities. In light of our findings, IST seems to be able to meet this need with its topoisomerase I enzyme inhibition activity. The determination of the interaction between the compound and topoisomerase enzymes indicates, IST has the possibility of being a topoisomerase enzyme inhibitor and therefore a potential anticancer agent. Our study revealed the interaction of Istaroxime with topoisomerases I and II through molecular docking analyzes and CDFT calculations. Consistent with the in silico results, ex vivo enzyme assays showed that IST could inhibit topoisomerase I, but decatenation analysis failed to demonstrate topoisomerase II inhibition in the presence of IST, as demonstrated by molecular docking analyses. We suggest that a more detailed analysis of the anticancer properties of IST is worth considering in anticancer drug research.

## 4. Materials and Methods

All of the compounds used in our experiments were HPLC-grade and were purchased in lyophilized form. Istaroxime (AdooQ, Irvine, CA, USA), Camptothecin, and Etoposide (Sigma-Aldrich, St. Louis, MO, USA) were dissolved in 100% Dimethyl Sulfoxide (DMSO). The MCF7 cell line was kindly gifted by Prof. Dilek Telci Temeltas, Genetics and Bioengineering Department of Yeditepe University. A549 (CCL-185) and PC3 (CRL-1435) cell lines were obtained from ATCC.

### 4.1. Cell Proliferation Assay

The effect of the IST was examined by a 3-(4,5-dimethylthiazol-2-yl)-2,5-diphenyl tetrazoliumbromide (MTT) (Sigma-Aldrich, St. Louis, MO, USA assay against the breast cancer cell line (MCF7), non-small lung cancer cell line (A549), and prostate cancer cell line (PC3). Each cell line was grown and maintained in DMEM (Gibco, Thermo Fisher Scientific, Waltham, MA, USA) medium containing 10% (*v*/*v*) heat-inactivated fetal bovine serum (FBS) (Gibco, Thermo Fisher Scientific, Waltham, MA, USA), 2 mM L-glutamine (Gibco, Thermo Fisher Scientific, Waltham, MA, USA), and 50 units/mL penicillin-streptomycin (Invitrogen, Thermo Fisher Scientific, Waltham, MA, USA). 1 mg/mL of MTT was applied to the cells. When MTT is applied to living cells, it transforms into a blue-violet, water-insoluble reduced form, formazan. The determination of viable cell number was calculated by determining the color intensity obtained after dissolving formazan in alcohol by photometric measurements. To investigate the effect of IST on MCF7, A549, and PC3 cell proliferation, IST was applied at 5 different concentrations (1.25–20 µM). The cells were seeded at a density of 3500 cells/well a day before treatment with the sample. Subsequently, different concentrations of IST were administered to cells. After 72 h of incubation, formazan formation was determined for each concentration, and formazan crystals were dissolved by the addition of 150 µL of isopropanol. The percentage of viable cells was calculated based on the values acquired by colorimetric methods using the Ascent spectrophotometer at 570 nm. Cell group which was not treated with IST was considered 100% viable (the control group), and its cytotoxic effect was calculated as follows: (Compound Abs − Blank Abs) × 100/(Control Abs − Blank Abs). Then the IC50 values of IST on each cell line was calculated using the curve fitting method. Each condition was studied in five replicates. 

### 4.2. Molecular Docking

The three-dimensional coordinates of Topoisomerases were accurately extracted from the Protein Data Bank (PDB) database [53]. The Istaroxime drug has undergone comprehensive evaluation for its potential interactions with specific protein structures, as detailed in Figure 2, and the corresponding docking scores, reflecting its affinity for these proteins. These investigations have unveiled the drug’s extraordinary promise as a robust candidate, with a distinct focus on targeting PDB ID: 4G0U [54] (Topoisomerase II) and 1T8I [55] (Topoisomerase I) structures. The interaction between this molecule and the target protein was subjected to a thorough and rigorous evaluation process. This evaluation encompassed the utilization of two key parameters: the docking score, which serves as an indicator of the affinity between the molecule and the protein, and the dG binding, which quantifies the binding energy. These evaluations were conducted with meticulous attention to detail, employing the advanced MMGBSA (Molecular Mechanics/Generalized Born Surface Area) methodology [56].

### 4.3. Enzyme Activity Tests

Supercoiled DNA relaxation and decatenation reactions were performed in enzyme activity assays. The reaction products were separated by horizontal electrophoresis (5 V/cm) in a 1 × TAE buffer on a 1% agarose gel. The reaction products of both the relaxation and decatenation tests were run on gel in the absence of EtBr and visualized under UV [14,40,49].

#### 4.3.1. Supercoiled DNA Relaxation Assays

Enzyme tests were performed with 1 unit of topoisomerase enzyme activity (Topogen, Buena Vista, CO, USA) relaxing 0.5 μg of supercoiled plasmid DNA (Takara, Shiga, Japan) in a reaction volume including 20 µL of total buffer at 37 °C for 30 min. The buffer content in which this analysis takes place is 72 mM KCl, 5 mM MgCl_2_, 5 mM DTT, 5 mM spermidine, and 0.1% bovine serum albumin (BSA). In line with the observability of the activity of topoisomerase enzymes in relaxation assays, as the enzyme relaxes the supercoiled DNA molecules, the DNA molecules in the relaxed form move as a band left behind in the agarose gel [41,57]. With the way the enzymes work at this point, topoisomerase enzyme activity is inhibited by the interaction of the inhibitor, and relaxed DNA molecules cannot be formed at the end of the reaction.

#### 4.3.2. Decatenation Assays

Topoisomerase II enzyme inhibitors can cleave and assemble small circular DNA molecules that are interlocked with each other, called cateneted DNA (kinetoplast DNA, kDNA, and Topogen, Buena Vista, CO, USA). As a result of these controlled temporary breaks, the separated DNA molecules are transformed into a less complex form, allowing them to walk through the agarose gel. Based on the structural differences between the DNA molecules formed by the interaction of an inhibitor molecule with the enzyme, it can be determined whether the enzyme activity is inhibited or not [41,58]. Decatenation assays were performed by incubating 0.2 μg of kinetoplast DNA (kDNA) and 1 unit of topoisomerase II enzyme in a final volume of 20 μL in reaction buffer (50 mM Tris-Cl (pH 8.0), 120 mM KCl, 10 mM MgCl_2_, 0.5 mM ATP, 0.5 mM DTT) for 30 min at 30 °C. Reaction products of topoisomerase II (Inspiralis, Norwich, UK) decatenation of catenated DNA substrates were analyzed in the presence and absence of the test compound by incubation at 37 °C for 30 min. Decatenation reactions were terminated using topoisomerase II stop buffer (5% sarcosyl, 0.0025% bromphenol blue, and 25% glycerol), followed by reaction products run on 1% TAE buffered agarose gel and visualized under UV.

### 4.4. Details of Density Functional Calculations

All Density Functional Theory computations, including geometry optimization, molecular orbital determination, and frequency analysis, were conducted at the B3LYP/6-311G** computational level and using GAMESS-US software [32]. We chose B3LYP because it is recognized as one of the most accurate functionals for small organic molecules [59,60] and is commonly used for drug modeling [61]. The effect of the DMSO solvent was taken into account with the polarized continuum model [62] implemented in GAMESS. In Table S1 of the Supplementary Material File, Atomic coordinates (Å) of the Istaroxime molecule optimized in the DMSO solvent at the B3LYP/6-311G** level of theory are presented, while Table S2 includes vibrational frequencies (cm^−1^) of the Istaroxime molecule calculated in the DMSO solvent at the B3LYP/6-311G** level of theory.

## Data Availability

Data are contained within the article and Supplementary Materials.

## Supplementary Materials

The following supporting information can be downloaded at: https://www.mdpi.com/article/10.3390/molecules28227458/s1, Table S1. Atomic coordinates (Å) of the Istaroxime molecule, optimized in DMSO solvent at the B3LYP/6-311G** level of theory. Table S2. Vibrational frequencies (cm^−1^) of the Istaroxime molecule, calculated in the DMSO solvent at the B3LYP/6-311G** level of theory.
