# Supplementary material for: Evaluation of the Anticancer and Biological Activities of Istaroxime via Ex Vivo Analyses, Molecular Docking and Conceptual Density Functional Theory Computations
Source: Molecules. 2023 Nov 7;28(22):7458. doi: 10.3390/molecules28227458 (PMC10672917; doi:10.3390/molecules28227458)
Supplement: Supplementary file 1 [file molecules-28-07458-s001.zip › molecules-2645752-supplementary.pdf]

Evaluation of the Anticancer and Biological Activities of Istaroxime via Ex Vivo Analyses, Molecular Docking and Conceptual Density Functional Theory Computations

Ege Gok [1], Naz Unal [2], Burcin Gungor [2], Gulderen Karakus [3], Savas Kaya [4,*], Pakize Canturk [1,*] and Konstantin P. Katin [5]

* Correspondence: savaskaya@cumhuriyet.edu.tr (S.K.); pcanturk@cumhuriyet.edu.tr (P.C.)

SUPPLEMENTARY MATERIALS

Table S1. Atomic coordinates (Å) of the Istaroxime molecule, optimized in DMSO solvent at the B3LYP/6-311G** level of theory.

C	1.71826	-7.11401	-0.29016
C	0.54426	-8.08202	-0.08940
C	0.35825	-8.34696	1.39307
C	1.76558	-8.15760	1.97441
C	2.43614	-7.06200	1.09573
C	4.62608	-6.44559	-0.13138
C	3.91035	-6.69870	-1.49643
C	2.67703	-7.60797	-1.38705
C	3.97135	-7.22703	1.02696
C	6.81145	-6.55168	1.19198
C	6.17220	-6.69601	-0.19554
C	4.59128	-6.80810	2.36665
C	6.07829	-6.62591	2.31671
C	8.84697	-5.42595	0.21772
C	8.31693	-5.57894	-1.17972
C	6.77643	-5.62227	-1.14459
C	8.32786	-6.41898	1.23065
N	9.66997	-4.54126	0.65092
O	10.13806	-3.67853	-0.36638
C	11.02080	-2.70293	0.20698
C	11.46941	-1.77962	-0.92050
N	10.40710	-0.99250	-1.56325
O	6.57758	-6.53988	3.59915
O	-0.13427	-8.55053	-0.99005
H	4.50834	-5.38082	0.11890
H	7.53973	-6.42781	3.59397
C	6.50192	-8.11618	-0.72978
H	4.18256	-8.29440	0.87751
H	2.22440	-6.08229	1.54430
C	1.10374	-5.74570	-0.66674
H	2.99230	-8.63538	-1.16175
H	2.15025	-7.64956	-2.34647
H	1.87245	-4.96697	-0.69159
H	0.62384	-5.79068	-1.65047
H	0.35062	-5.43286	0.06626
H	-0.33158	-7.57956	1.77618
H	-0.09946	-9.32277	1.57829
H	1.75365	-7.89086	3.03485
H	2.32549	-9.09814	1.88394
H	3.61338	-5.74033	-1.93469
H	4.59233	-7.14636	-2.22334
H	4.14189	-5.86268	2.70765
H	4.36138	-7.54722	3.14440
H	8.71229	-6.12777	2.21405
H	8.78949	-7.39422	1.00611
H	6.41018	-5.78156	-2.16477
H	6.41358	-4.63555	-0.82893
H	7.57310	-8.32952	-0.67082
H	5.99475	-8.88664	-0.14123
H	6.21515	-8.24355	-1.77845
H	8.65931	-4.76388	-1.81909
H	8.71374	-6.51233	-1.60213
H	10.49068	-2.13986	0.98867
H	11.88551	-3.20615	0.66205
H	12.22035	-1.09200	-0.51361
H	11.96824	-2.37792	-1.69331
H	9.68560	-1.61319	-1.92742
H	9.94663	-0.38916	-0.88302

Table S2. Vibrational frequencies (cm^{-1}) of the Istaroxime molecule, calculated in the DMSO solvent at the B3LYP/6-311G** level of theory.

# of mode	frequency	# of mode	frequency	# of mode	frequency
1	24.44	57	889.00	113	1390.90
2	48.81	58	893.79	114	1395.88
3	50.94	59	920.09	115	1398.64
4	58.24	60	921.33	116	1403.06
5	64.10	61	936.75	117	1404.42
6	75.03	62	945.57	118	1414.76
7	100.68	63	952.47	119	1439.91
8	116.49	64	962.83	120	1459.96
9	126.45	65	973.92	121	1461.91
10	139.22	66	981.39	122	1468.40
11	151.29	67	988.89	123	1470.48
12	168.43	68	1006.01	124	1472.55
13	173.19	69	1014.28	125	1480.34
14	198.63	70	1019.75	126	1486.61
15	212.01	71	1030.74	127	1487.53
16	224.57	72	1039.33	128	1490.03
17	230.90	73	1042.45	129	1494.29
18	239.52	74	1057.09	130	1496.03
19	253.30	75	1074.81	131	1500.92
20	263.32	76	1078.47	132	1507.58
21	285.00	77	1087.00	133	1642.08
22	295.39	78	1092.53	134	1702.03
23	307.68	79	1099.18	135	1723.90
24	321.91	80	1120.89	136	1773.42
25	331.39	81	1128.60	137	2974.64
26	346.58	82	1136.85	138	2984.18
27	351.90	83	1145.44	139	2990.21
28	356.90	84	1166.38	140	2995.13
29	370.43	85	1175.64	141	3006.36
30	396.03	86	1186.23	142	3009.08
31	409.25	87	1198.69	143	3014.16
32	417.29	88	1204.31	144	3015.46
33	433.20	89	1210.41	145	3018.77
34	467.79	90	1221.01	146	3020.48
35	473.62	91	1229.06	147	3021.27
36	500.73	92	1237.10	148	3022.47
37	507.81	93	1239.34	149	3024.65
38	525.33	94	1256.29	150	3031.05
39	535.07	95	1265.24	151	3036.55
40	549.24	96	1267.14	152	3042.21
41	575.72	97	1273.25	153	3045.38
42	588.59	98	1280.95	154	3058.16
43	611.63	99	1302.73	155	3064.36
44	623.51	100	1307.60	156	3066.62
45	645.24	101	1314.93	157	3069.77
46	662.71	102	1324.41	158	3080.66
47	687.92	103	1333.79	159	3084.65
48	716.42	104	1337.12	160	3088.08
49	736.51	105	1342.76	161	3093.32
50	776.65	106	1349.74	162	3095.15
51	812.39	107	1354.27	163	3098.65
52	821.01	108	1367.34	164	3115.89
53	828.03	109	1372.82	165	3118.81
54	835.77	110	1377.53	166	3472.82
55	874.38	111	1384.60	167	3543.60
56	881.13	112	1388.24	168	3792.47
